# Full-Length Hairpin RNA Accumulates at High Levels in Yeast but Not in Bacteria and Plants

**DOI:** 10.3390/genes10060458

**Published:** 2019-06-15

**Authors:** Chengcheng Zhong, Neil A. Smith, Daai Zhang, Simon Goodfellow, Ren Zhang, Weixing Shan, Ming-Bo Wang

**Affiliations:** 1 State Key Laboratory of Crop Stress Biology for Arid Areas, Northwest A&F University, Yangling 712100, China; zcc_1127@hotmail.com; 2 Commonwealth Scientific and Industrial Research Organisation (CSIRO) Agriculture and Food, Canberra, ACT 2601, Australia; neil.smith@csiro.au (N.A.S.); anna.zhang@csiro.au (D.Z.); 3 College of Plant Protection, Northwest A&F University, Yangling 712100, China; 4 School of Chemistry and Molecular Bioscience, University of Wollongong, NSW 2522, Australia; sg389@uowmail.edu.au (S.G.); rzhang@uow.edu.au (R.Z.); 5 College of Agronomy, Northwest A&F University, Yangling 712100, China

**Keywords:** hairpin RNA, double-stranded RNA, RNA interference, yeast

## Abstract

Hairpin-structured (hp) RNA has been widely used to induce RNA interference (RNAi) in plants and animals, and an in vivo expression system for hpRNA is important for large-scale RNAi applications. Bacterial expression systems have so far been developed for in vivo expression of hpRNA or double-stranded (ds) RNA, but the structure of the resulting RNAi molecules has remained unclear. Here we report that long hpRNAs expressed in the bacteria *Escherichia coli* and *Sinorhizobium meliloti* were largely processed into shorter dsRNA fragments with no or few full-length molecules being present. A loss-of-function mutation in the dsRNA-processing enzyme RNase III, in the widely used *E. coli* HT115 strain, did not prevent the processing of hpRNA. Consistent with previous observations in plants, the loop sequence of long hpRNA expressed in *Agrobacterium*-infiltrated *Nicotiana benthamiana* leaves was excised, leaving no detectable levels of full-length hpRNA molecule. In contrast to bacteria and plants, long hpRNAs expressed in the budding yeast *Saccharomyces cerevisiae* accumulated as intact, full-length molecules. RNA extracted from hpRNA-expressing yeast cells was shown to be capable of inducing RNAi against a β-glucuronidase (GUS) reporter gene in tobacco leaves when applied topically on leaf surfaces. Our results indicate that yeast can potentially be used to express full-length hpRNA molecules for RNAi and perhaps other structured RNAs that are important in biological applications.

## 1. Introduction

Hairpin-structured RNA (hpRNA) is widely used to induce RNA interference (RNAi) in plants and animals, where the long double-stranded RNA (dsRNA) is processed by Dicer or Dicer-like (DCL) proteins into 21–24 nt small interfering RNAs (siRNAs) that bind to and guide Argonaute proteins to degrade cognate single-stranded RNA [1]. In plants, RNAi has been achieved mainly through transgenic expression of long hpRNA [2], but, recently, exogenous applications of hpRNA or dsRNA have been actively exploited to induce RNAi against plant genes or cross-kingdom RNAi against plant pest and pathogen genes [3].

Exogenous RNAi was first demonstrated in *Caenorhabditis elegans* where ingestion of synthetic dsRNA or bacteria expressing dsRNA triggered systemic gene silencing [4,5]. In plants, increasing evidence in recent years has indicated that sufficient gene silencing can be triggered in insect pests by uptake of dsRNA molecules from artificial diets or host plants [6,7,8,9,10]. For instance, transgenic corn [11] or potato [12] plants engineered to express hpRNAs against coleopteran insect genes showed significant insect resistance. The efficiency of exogenous or cross-kingdom RNAi depends on the high amount of long dsRNAs [12,13,14]. In vitro bidirectional transcription using T7 polymerase from DNA fragments has been widely used to synthesize dsRNA [6,11,15,16], but such an in vitro system is not suited for large-scale production of dsRNA. An *Escherichia coli* strain HT115, defective in RNase III activity and carrying an inducible T7 RNA polymerase cassette in its genome, has been developed to express dsRNA [4,5,17]. Applying HT115-expressed dsRNA or hpRNA of viral sequences onto plant leaves has been shown to confer resistance to virus infections [7,18,19,20,21]. Recently, a bacteriophage phi6 system was developed for in vivo dsRNA production in *Pseudomonas syringae* bacteria by taking advantage of the strategies of phi6 infection, and dsRNA expressed using this system was shown to provide protection against tobacco mosaic virus infection when applied topically to tobacco plants [22]. However, while these bacterial expression systems can potentially be used for low-cost production of dsRNA, the integrity of the resulting dsRNA structures has not been investigated.

Yeasts are eukaryotic, single-cellular organisms belonging to the kingdom of fungi, and they have long been used in food processing, drug discovery, and gene expression studies. Recently, yeasts have also been widely used in virus research. Single-stranded RNA viruses hosted in plants or animals can be replicated in the budding yeast *Saccharomyces cerevisiae* [23,24,25,26]. Avocado sunblotch viroid (ASBVd), a plant subviral RNA pathogen with a highly structured genome, is demonstrated to undergo proper self-cleavage and replicate in *S. cerevisiae* [27]. A unique feature of *S. cerevisiae* among fungi and other eukaryotic organisms is the lack of critical components for RNAi such as Dicer, Argonaute, and RNA-dependent RNA polymerase (RdRP) [28].

In the present study we found that long hpRNA expressed in the bacteria *E. coli* and *Sinorhizobium meliloti* was processed into shorter dsRNA molecules. This processing of hpRNA also occurred in the RNase III-deficient *E. coli* strain HT115, indicating that the bacterial expression system was not suitable for generating structured RNA molecules such as long hpRNA. In contrast to bacteria, we showed that long hpRNA expressed in *S. cerevisiae* cells remained intact, making yeast a potential system for large-scale production of hpRNA and other structured RNA molecules.

## 2. Materials and Methods

### 2.1. Plasmid Constructs

The long β-glucuronidase (GUS) hpRNA construct (hpGUS-1) used in *Nicotiana benthamiana Agrobacterium* infiltration experiment (pMBW306) was described previously by Shen et al [29], and the sequence of hpGUS-1 RNA is shown in the Supplementary File. To construct the two plasmids used for expression in *Sinorhizobium meliloti*, pMBW306 was firstly modified by adding a tetracycline resistance marker gene (*tetA*) to make the p35S construct and followed by inserting a *S. meliloti* tyrosine tRNA promoter [30] to make the pSm construct. T7 promoter-driven hpGUS-1 constructs were made by excising the hpGUS-1 sequence from the p35S-hpGUS-1-OCS-T cassette in pMBW281 (Supplementary File) using *Nco*I digestion, which was inserted (through Pfu DNA polymerase-treated blunt end ligation) into the *Eco*RI site of a plasmid with a T7 promoter and T7 terminator (pBact-term-JKK, Supplementary Data). The *E. coli* HT115 strain [17] and *S. meliloti* 1021 strain were kindly provided by Dr. Peter Boag (Monash University, Australia) and Dr. Michael Djordjevic (ANU, Australia), respectively. They were transformed using electroporation, and the transformants were cultured at 37 °C (*E. coli*) or 30 °C (*S. meliloti*) in liquid Luria-Bertani (LB) medium containing appropriate antibiotics.

Three yeast expression constructs were built in the pGADT7 vector (kindly provided by Peter Dodds, CSIRO, Australia). The yeast hpGUS-1 construct was prepared by inserting the hpGUS-1 sequence (*Nco*I fragment) from pMBW306 into the *Hin*dIII site of pGADT7 (digested DNA ends blunted using Pfu DNA polymerase before ligation). Similarly, hpGUS-2 and hpGFP sequences (Supplementary File) were excised from p35S-hpRNA-OCS-T cassettes using *Kpn*I/*Hin*dIII and inserted into pGADT7 at the *Hin*dIII site through Pfu polymerase-treated blunt end ligation to form yeast expression vectors for hpGUS-2 and hpGFP.

### 2.2. Yeast Transformation

The HF7c yeast strain was used for transformation with the three yeast expression constructs, following the modified protocol outlined in the yeast protocols handbook (Clontech). For preparing HF7c competent cells, the culture was adjusted to OD_600_ = 0.4–0.8, the cells were pelleted and washed with 100 mM lithium acetate, and then the cells were resuspended in 100 mM lithium acetate. Plasmid DNA (500 ng) and 320 µL transformation solution (240 µL 50% PEG 3350, 33 µL 1M lithium acetate, and 50 µL 2 mg/mL boiled salmon sperm DNA) were added into 50 µL of HF7c competent cells, followed by vortexing. The mixture was incubated at 30 °C for 30 min then at 42 °C for 30–45 min with gentle inversion every 10 min. The cells were pelleted and resuspended in 200 µL sterile water and gently plated on selective leucine-free medium (6.72g/L yeast nitrogen base without amino acids, 1.4 g/L dropout supplement, 2% glucose, 200 mg/L histidine, 200 mg/L tryptophan, and 200 mg/L uracil). Colonies appeared after 2–3 d incubation at 28 °C.

### 2.3. Agrobacterium Infiltration of *Nicotiana benthamiana* Leaves

*Agrobacterium* infiltration with pMBW306 (35S-hpGUS-1) was performed as previously described including a green fluorescence protein (GFP)-expressing construct as visual marker and a construct encoding the Cucumber mosaic virus 2b protein for enhancing gene expression [29]. Infiltrated tissues were harvested 3 d post infiltration and used for RNA isolation. 

### 2.4. Northern Blot Analysis and qRT-PCR

Total RNA was extracted using Trizol Reagent (Invitrogen, #15596026, Carlsbad, CA, USA) following the manufacturer’s instructions. For the transformants of yeast and bacteria, cultures were grown with appropriate antibiotic selection until OD_600_ = 0.8, and the cells were pelleted and resuspended in Trizol Reagent. 

Northern blot hybridization was performed essentially as described using the same hybridization solutions and procedures [31]: 50 ng of in vitro transcribed RNA and 8 µg of total RNA from plants, yeasts, or bacteria were separated in a 1.3% formaldehyde agarose gel and then transferred to Hybond-N membrane (GE Healthcare Amersham, #RPN203N, Pittsburgh, PA, USA). After UV cross-linking, the membrane was prehybridized at 55 °C for 2–4 h, then it was hybridized overnight with an antisense full-length GUS probe incorporated with [α−^32^P] UTP by in vitro transcription [31]. The hybridized membranes were washed three times with 40 mM sodium phosphate buffer (pH = 7.2) containing 1% SDS and 1 mM EDTA for 15 min per wash at 65 °C, and they were visualized on a phosphorimager. 

For qRT-PCR analysis of the stem and loop expression, 2 µg of total RNA isolated from yeast (expressing hpGUS-1) and HT115 (containing the 35S-driven hpGUS-1) was treated with RNase-free DNase I (Ambion, #AM2222, Naugatuck, CT, USA) at 37 °C for 30 min, then it was purified with phenol/chloroform extraction and ethanol precipitation. The purified RNA was reverse transcribed with Superscript III reverse transcriptase (Invitrogen) following the manufacturer’s instructions using random hexamer primers. Real-time PCR was performed in three technical triplicates on a Corbett 2000 Rotor-Gene machine (Corbett Research, Northampton, MA, USA), using Fast SYBR Green Master Mix (Applied Biosystems, #4385610, Foster, CA, USA), following the manufacturer’s instructions. The same RNA samples (2 µg) without DNase treatment and reverse transcription were amplified using the same primer pairs for use as reference for qPCR normalization. Primer sequences are listed in the Supplementary File.

### 2.5. Exogenous RNAi Assay

A homozygous, transgenic tobacco (*Nicotiana tabacum* Wisconsin 38) line containing a single copy of constitutively expressed GUS gene (MB Wang, unpublished) was used for the RNAi assay. Expanded tobacco leaves were excised from plants in a growth room and placed in a Petridish on top of a wet filter paper. Each leaf was cut into three sections to avoid cross contamination among the three hpRNAs, and an approximately 2 cm^2^ area of each section was treated with hpRNA samples. Sixty micrograms of yeast or bacteria total RNA in 60 µL resuspension buffer (100 mM KCl, 62.5 mM HEPES, 0.05% Silwet L-77) was applied to the leaf areas using a soft paintbrush, usually requiring more than one brushing to apply the whole 60 µL solution. The treated leaf sections were kept in a petri dish at room temperature under natural light. The same assay was performed on four separate GUS-expressing leaves. 

### 2.6. Analysis of β-glucuronidase (GUS) Silencing Using a 4-Methylumbelliferyl-β-d-Glucuronide (MUG) Assay

hpRNA-treated leaf samples were harvested by cutting out half of the treated areas using a pair of scissors at 5 and 22 h after treatment, and they were immediately frozen in liquid nitrogen. A 4-methylumbelliferyl-β-d-glucuronide (MUG) assay was performed using the kinetic method as described in Chen et al [32]. Basically, leaf tissues were ground to powder in liquid nitrogen using a mortar and pestle, resuspended in protein extraction buffer (50 mM NaPO_4_, pH = 7; 10 mM EDTA; 0.1% Triton X-100; 0.1% *N*-laurylsarcosine; and 10 mM β-mercaptoethanol), and centrifuged at 4 °C for 10 min. Supernatant was transferred into a fresh Eppendorf tube, and 5 µL was used for each measurement. MUG assay was carried out in three technical replicates at 37 °C, and the relative GUS activity was calculated using the slope of the reaction curve against protein concentration [32]. 

## 3. Results

### 3.1. Long hpRNA Expressed in Escherichia coli and Sinorhizobium meliloti Is Processed into Short Fragments

**Figure 1:**
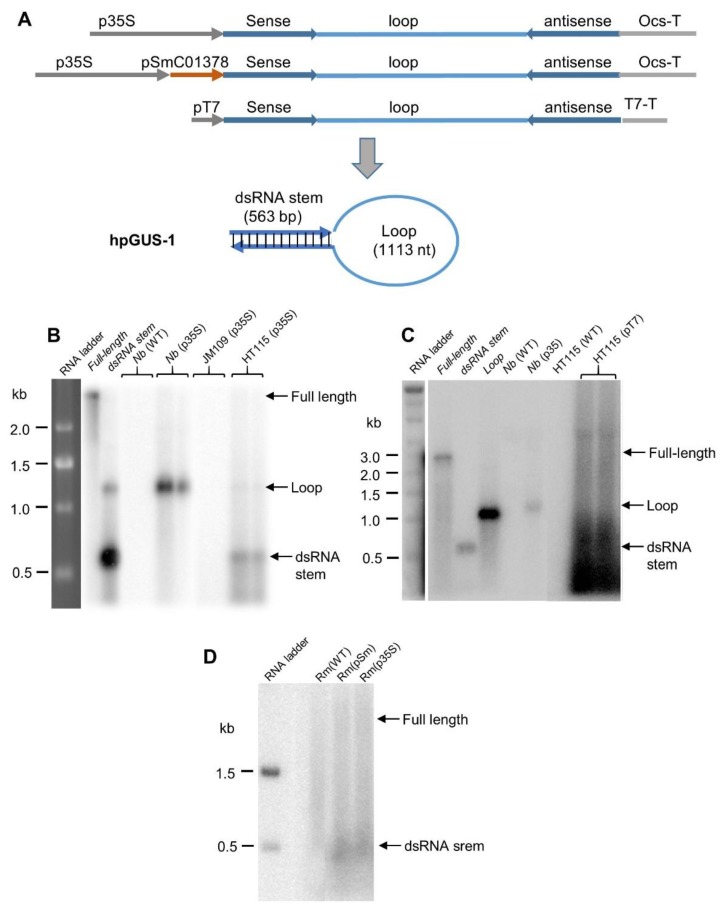
Long hpRNA expressed in plants and bacteria is processed into short fragments. (**A**) Schematic of the β-glucuronidase (GUS) hpRNA constructs and predicted hpRNA structure. p35S and pSmC01378 are the cauliflower mosaic virus 35S RNA promoter and *Sinorhizobium meliloti* tRNA promoter, respectively. (**B**) Northern blot hybridization showing that GUS hpRNA expressed by the 35S promoter in *Agrobacterium*-infiltrated *Nicotiana benthamiana* (Nb) leaves and the RNase III-deficient *Escherichia coli* strain HT115 were processed into the loop or dsRNA stem. Eight micrograms of total RNA were separated in a 1.3% formaldehyde agarose gel, transferred to a Hybond-N membrane, and hybridized with ^32^P-labeled full-length antisense GUS RNA. Almost no signals were detected in the wild-type *E. coli* strain JM109 background, confirming the advantages of HT115 for dsRNA accumulation. (**C**) GUS hpRNA expressed by the T7 polymerase promoter in *E. coli* HT115 was processed into short RNA fragments. (**D**) GUS hpRNA expressed by two different promoters in *Sinorhizobium meliloti* (Sm) strains was processed into short RNA fragments. The “Full-length”, “Loop”, and “dsRNA Stem” in the left lanes of (**B**,**C**) are in vitro transcripts (50 ng each), where the dsRNA stem was prepared by annealing sense and antisense transcripts.

The RNase III-deficient *E. coli* strain HT115 was developed to facilitate the production of dsRNA for RNAi, which allows for a high level of dsRNA accumulation because of the loss of dsRNA-specific RNase III activity [4,5,17]. It has been widely used for expressing dsRNA or hpRNA for RNAi experiments [4,7,18,19,20,21], but it was unclear if the long hpRNA accumulated as an intact molecule. We first introduced into wild-type *E. coli* and HT115 a plasmid construct designed to express long hpRNA (hpGUS-1) with a ~560 bp dsRNA stem plus ~1.1 kb loop derived from the coding sequence of the β-glucuronidase (GUS) gene. This construct was driven by a cauliflower mosaic virus 35S promoter (Figure 1A). The 35S promoter is known to have transcriptional activity in bacteria [33], and the 35S-hpGUS-OCS cassette in Figure 1A is the same as the one used for *Agrobacterium* infiltration of *Nicotiana benthamiana* leaves in the current study and in Shen et al. [29]. As shown in Figure 1B, the strongest RNA band detected in the HT115 samples corresponded to the dsRNA stem, above the 500 nt RNA size marker. Another larger sized band, of relatively low abundance, corresponded to the loop fragment of the GUS hpRNA. Accumulation of such loop fragments from hpRNA was previously observed in transgenic *Arabidopsis* plants [34] and in *Agrobacterium*-infiltrated *N. benthamiana* leaves [29]. Indeed, the loop fragment was clearly detected in total RNA isolated from *N. benthamiana* leaves infiltrated with *Agrobacterium* carrying the same GUS hpRNA construct (Figure 1B). Unlike the dsRNA stem and loop signals, almost no full-length GUS hpRNA fragments could be detected in the northern blot from the *E. coli* or the *N. benthamiana* samples. The bacterial phage T7 DNA polymerase promoter is usually used to express dsRNA or hpRNA in HT115, as this strain contains a T7 polymerase expression cassette in the genome [4,35]. We, therefore, prepared a T7 promoter construct expressing the same long GUS hpRNA (Figure 1A) and introduced this construct into HT115. Northern blot hybridization again showed that no distinct full-length GUS hpRNA band could be detected, with the majority of the hybridizing signals located around or below the position of dsRNA stem (Figure 1C).

To test hpRNA accumulation in another bacterial species, we introduced the 35S and a *Sinorhizobium meliloti* tRNA promoter-driven constructs encoding the same GUS hpRNA (Figure 1A) into *S. meliloti*, and we analyzed hpRNA accumulation using northern blot hybridization (Figure 1D). Similar to the pattern of RNA signals in HT115 containing the T7-driven construct, the majority of GUS-hpRNA-derived signals were located around the position of dsRNA stem size, and no full-length molecule could be detected. Thus, long hpRNA was processed and did not accumulate as an intact molecule in either *E. coli* or *S. meliloti*.

### 3.2. hpRNA Expressed in Yeast Cells Accumulates as a Full-Length Molecule

**Figure 2:**
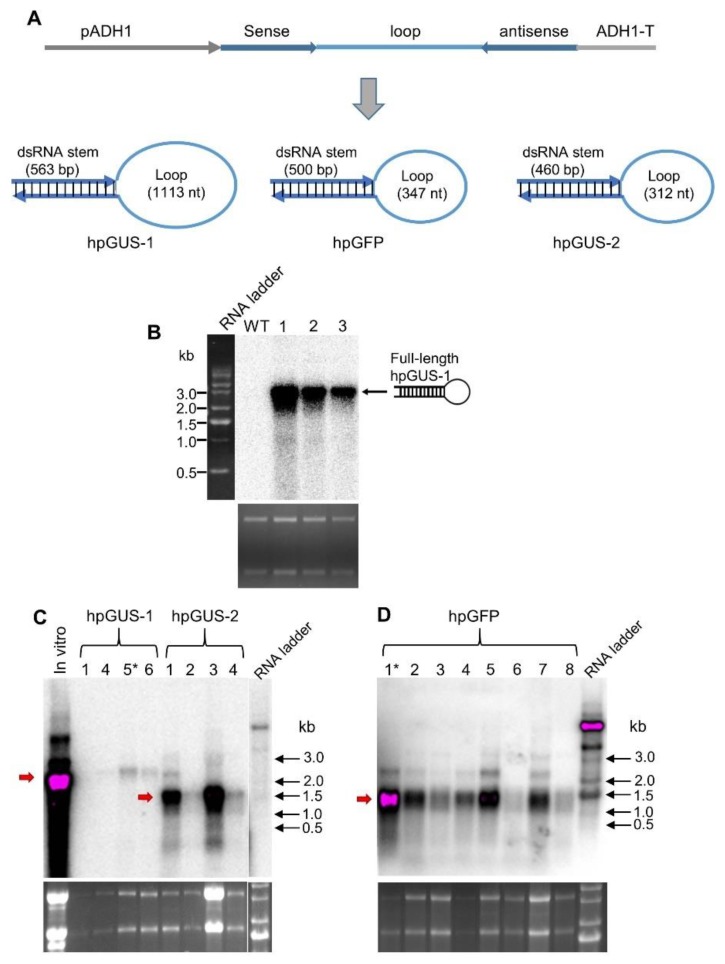
hpRNA expressed in yeast cells accumulates as an intact molecule. (**A**) Schematic diagram of the yeast expression vectors and the predicted structure of the hpRNAs. pADH1 and ADH1-T are yeast *Alcohol Dehydrogenase 1* gene promoter and 3’ transcriptional terminator, respectively. (**B**) Only full-length GUS hpRNA-1 was detectable in three independent transgenic yeast lines. (**C**) GUS hpRNA-2 also accumulated predominantly as full-length molecules in four independent transgenic yeast lines. Compared to hpGUS-1 (left four lanes), this shorter hpRNA accumulated at higher levels (right 4 lanes). (**D**) hpGFP RNA accumulated predominantly as full-length molecules in eight independent yeast lines. The red arrow indicates the position of the full-length hpRNAs. The asterisks indicate the transgenic lines of which total RNA extracts were used in gene silencing assay shown in Figure 4. For all three northern blots, 8 µg of total RNA was loaded for each sample and hybridized with ^32^P-labeled full-length antisense GUS RNA. The lower panels are ethidium bromide-stained RNA gels used as loading reference.

We inserted the same GUS hpRNA construct into a yeast expression vector (Figure 2A), and we transformed the construct into *S. cerevisiae* yeast. Total RNA from transformed yeast cells was analyzed by northern blot hybridization (Figure 2B). In contrast to RNA expressed in bacterial cells, no processed dsRNA stem or loop fragments was visible on the northern blot. Instead, a strong hybridizing band corresponding to the full-length hpRNA was detected (slightly larger than the 2.24 kb size of the in vitro hpGUS-1 transcript, presumably from the presence of polyadenylation tails). We introduced another construct encoding a shorter version of GUS hpRNA and a construct expressing hpRNA of the GFP coding sequence (Figure 2A) into yeast. As shown in Figure 2C,D, full-length hpRNA accumulated as the predominant RNA species in the transgenic yeast lines for both hpRNA constructs. These results indicated that the bulk of the hpRNA transcript was not processed in yeast and remained as a stable, full-length molecule.

**Figure 3:**
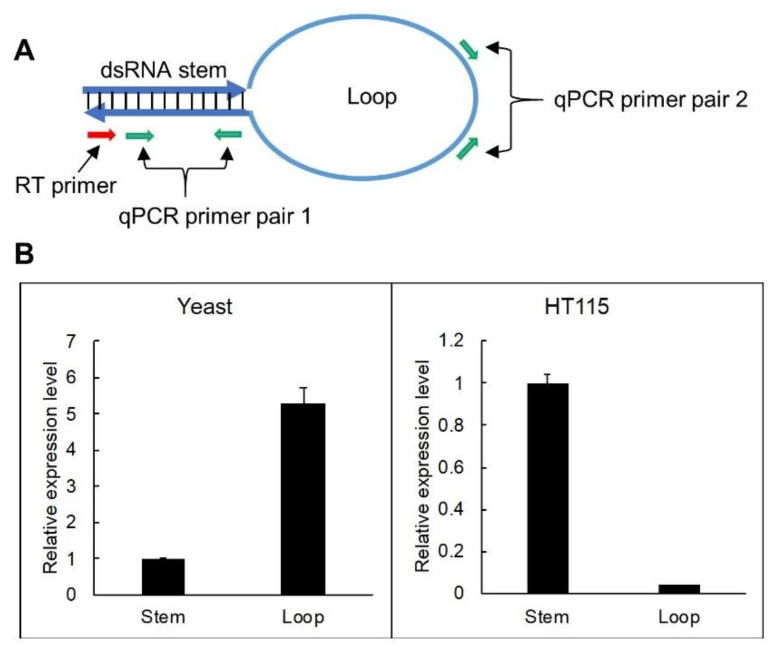
RT-qPCR detects a higher proportion of loop sequence in yeast than in *E. coli*. (**A**) Locations of RT-PCR primers. (**B**) RT-PCR results showing a higher proportion of amplification of the loop sequence vs dsRNA stem region in yeast than in *E. coli*. Total RNA samples (2 µg) from yeast (expressing hpGUS-1) and HT115 (containing the 35S-driven hpGUS-1) were analyzed, with three technical replicates for the qPCR reaction. The same RNA samples (2 µg) without DNase treatment and Superscript III reverse transcription were used as reference for qPCR normalization. Note that the value “1” for stem amplification is arbitrary, and the abundance of yeast and HT115-expressed dsRNA cannot be compared using this data because there is no common reference gene.

In order to verify the difference in hpRNA accumulation between bacteria and yeast, we performed RT-qPCR analyses to determine the ratio of full-length hpRNA in the two systems. Basically, reverse transcription (RT) was performed using a primer against the stem (red arrow, Figure 3A), which should be extended through to the loop if hpRNA is intact or terminate at the end of the stem if the loop is excised out of the hpRNA. Real-time PCR was then performed on the stem (primer pair 1) and loop (primer pair 2) sequences, respectively, which should detect both the stem and loop if hpRNA was full-length or only the stem if the loop was excised (Figure 3A). It should be noted that we did not try to amplify full-length hpRNA because our previous attempts to PCR amplify such long stem loop-structured sequences as a whole molecule were not successful (data not shown), possibly because DNA polymerase was unable to enter the double-stranded stem region of a stem loop structure from the single-stranded loop area. As shown in Figure 3B, RT-qPCR detected a relatively high ratio of dsRNA stem from RNA expressed in *E. coli* compared to the low ratio of loop product. In contrast, a relatively high ratio of loop RNA was detected from yeast RNA. This result indicated that reverse transcription was efficiently extended to the loop from the dsRNA stem for yeast-expressed hpRNA but not for *E. coli*-expressed RNA, confirming that yeast-derived hpRNA remained full-length, but *E. coli*-derived hpRNA was processed. The stronger amplification of loop than dsRNA stem from the yeast RNA likely was due to better RT-PCR amplification efficiency by the loop-specific primer pairs.

### 3.3. hpRNA Expressed in Yeast Is Effective at Inducing Gene Silencing through Topical Application

**Figure 4:**
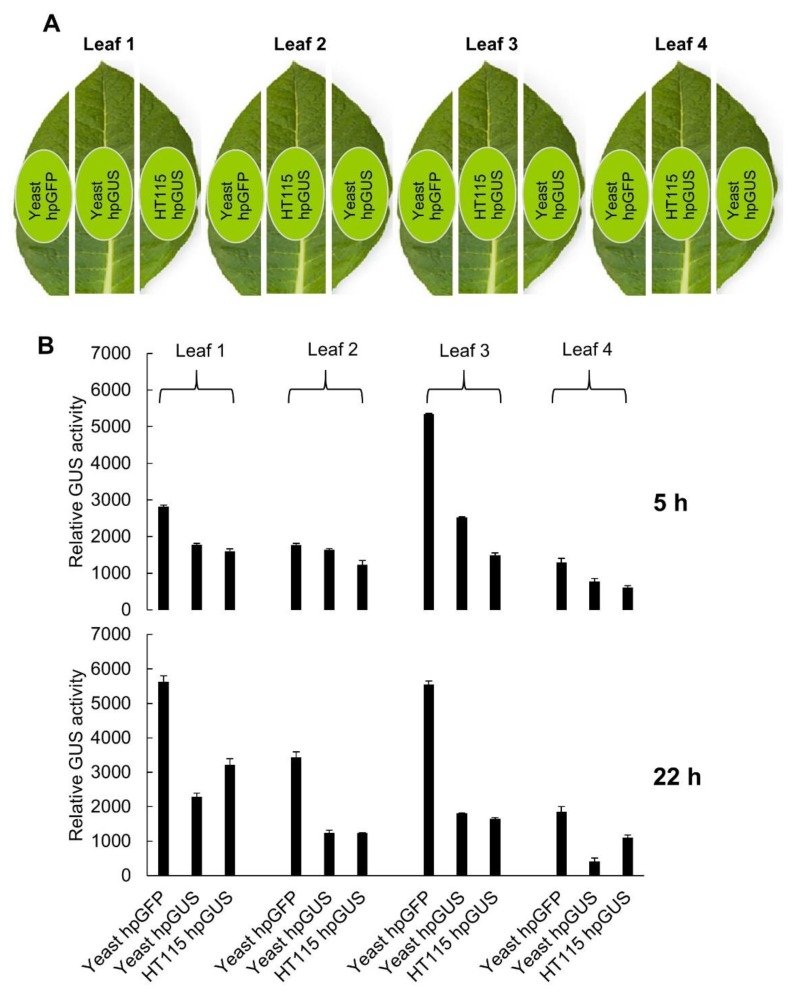
hpRNA expressed in yeast can induce exogenous RNAi against the GUS reporter gene in tobacco leaves. hpGFP RNA from yeast and hpGUS RNA from HT115 containing the 35S-driven hpGUS-1 construct were used as controls. (**A**) Illustration of three cut leaf sections, of which a ~2 cm^2^ area in the middle (highlighted in light green) was brushed with the three different RNA samples as specified. (**B**) Relative GUS activity measured using 4-methylumbelliferyl-β-d-glucuronide (MUG) assay with three technical replicates for each sample. Note that the relative effect on GUS expression of yeast derived RNA over HT115-derived RNA increased from the 5 h time point to the 22 h time point. The same assay was performed on four separate leaves.

To test the function of yeast-expressed hpRNA in RNAi, we extracted total RNA from the yeast hpGUS-1 Line #5 (shown in Figure 2C) and used the total RNA to treat detached tobacco leaves containing a highly-expressed GUS gene. Total RNA from GFP hpRNA-expressing yeast Line #1 (Figure 2D) was used as a nonspecific hpRNA control, and total RNA from *E. coli* HT115 with the 35S promoter-driven hpGUS-1 (used in the RT-qPCR analysis in Figure 3) was included for comparison. Sixty micrograms of total RNA in 60 µL suspension solution, containing approximately 100 ng of hpGUS-1 or hpGFP RNA (estimated based on northern blot hybridization using in vitro transcripts as reference), was brushed onto an approximately 2 cm^2^ area of tobacco leaf sections excised from a tobacco line constitutively expressing the GUS gene (Figure 4A). Half of the treated leaf area was harvested at 5 h and the other half at 22 h after hpRNA treatment, and it was assayed for GUS activity. As shown in Figure 4B, downregulation of GUS activity was induced in four separate leaves by RNA extracts from both yeast and HT115 cells expressing hpGUS-1 RNA. Interestingly, GUS hpRNA from the yeast line induced weaker GUS downregulation than hpRNA from HT115 at 5 h post treatment, but at 22 h the levels of GUS downregulation by the yeast-derived RNA became either the same as or greater than those by the HT115-expressed RNA (60%–80% downregulation by yeast RNA compared to 40%–70% by HT115 RNA). This result could suggest that the hpRNA structure of the full-length hpRNA from yeast was more stable and lasted longer than the processed, open-ended dsRNA stem structure of the HT115-derived RNA.

## 4. Discussion

In this study we demonstrated that long hairpin-structured RNA expressed in bacteria, including the RNase III-deficient *E. coli* strain HT115, was processed into short dsRNA fragments. HT115 has been widely used to express dsRNA for exogenous RNAi experiments, and in some cases the dsRNA was expressed through hpRNA constructs [18,20,21]. The structure of the resulting dsRNAs in HT115 was not investigated in these previous studies, and we initially assumed that hpRNA could accumulate in HT115 as intact molecules. Our results showed that in both *E. coli* HT115 and *S. meliloti* bacteria, the long hpRNA transcript was processed into short fragments, with the dominant detectable RNA species corresponding to the dsRNA stem. This suggested that the single-stranded loop of an hpRNA was prone to degradation in bacterial cells, and that the loss of RNase III activity cannot prevent this processing of loop sequences. Therefore, while bacteria can be used to express simple sense/antisense annealed dsRNA (or open-ended dsRNA), they are not suitable for expressing full-length hairpin-loop dsRNA molecules. Our results in this study also confirm the previous observation that hpRNA expressed in plants gives rise to a distinct fragment of the loop sequence, with little full-length hpRNA molecules detectable [29,34]. 

In contrast to bacteria, long hpRNA expressed in the laboratory yeast strain of *S. cerevisiae* accumulated mainly as full-length molecules. Yeasts are eukaryotic organisms encoding various RNases, including RNase III-like enzymes, in their genomes. How hpRNA loops are protected from RNase processing in *S. cerevisiae,* and whether this protection also occurs generally in other yeast strains or fungi, requires further investigation. A unique feature of *S. cerevisiae* among most of the eukaryotic organisms including fungi is that it lacks the key RNAi components such as Dicer [28]. It will be interesting to examine if the lack of RNAi components contributes to the protection of full-length hpRNA; however, hpRNA is processed in bacteria that also lack RNAi components, which implies that other RNA metabolizing pathways are involved. Regardless of the molecular mechanisms, our results demonstrated that *S. cerevisiae* can be used to express full-length hpRNA, and the resulting hpRNA was functional at inducing exogenous RNAi. Potentially, the yeast expression system can be modified for large-scale production of hpRNA or other structured RNAi molecules.

Currently, the amount of dsRNA from the yeast expression system using the yeast *ADH* gene promoter construct was relatively low, similar to that from HT115 cells expressing the 35S promoter-driven construct and well below the amount of T7 promoter-expressed dsRNA in HT115. We estimated using northern blot hybridization that each µg of yeast total RNA contained about 2 ng of hpRNA, whereas the bulk of RNA from the HT115-T7 polymerase system was hpRNA-derived product. The advantage of the yeast expression system is the integrity of the resulting structured RNA, and future improvements incorporating high-level expression cassettes similar to the T7 [4,35] or phi6 [22] polymerase systems would make the yeast system particularly useful for RNAi applications requiring the use of full-length RNAi molecules. While yeast cannot be used to directly express 20–25 nt siRNAs because of the lack of Dicer and other RNAi components, long hpRNA derived from yeast can be subsequently processed into siRNAs using commercially available RNase III enzyme for siRNA-directed exogenous RNAi. Alternatively, yeast can potentially be genetically modified to express RNAi machinery for direct expression of siRNAs.

In addition to exogenous RNAi, expression of structured RNAs is important in many other applications. For instance, viral and subviral RNAs are characterized by specific secondary structures that are important for their replication in host cells, and maintaining structural integrity is required for the infectivity of viral RNA transcripts in infection experiments [36]. Interestingly, *S. cerevisiae* has been widely used in virus studies, and a number of plant and animal RNA viruses and subviral RNAs have been shown to replicate successfully in the yeast cells [23,24,25,26,27]. It will be interesting to investigate if highly structured viral genomic RNAs and other biologically active RNAs can accumulate as full-length molecules in *S. cerevisiae*. If so, this yeast expression system can be widely used for the production of structured RNAs.

## Supplementary Materials

The following are available online at https://www.mdpi.com/2073-4425/10/6/458/s1.
