# Supplementary material for: Full-Length Hairpin RNA Accumulates at High Levels in Yeast but Not in Bacteria and Plants
Source: Genes (Basel). 2019 Jun 15;10(6):458. doi: 10.3390/genes10060458 (PMC6627737; doi:10.3390/genes10060458)
Supplement: Supplementary file 1 [file genes-10-00458-s001.pdf]

Full-length hairpin RNA accumulates at high levels in yeast but not in bacteria and plants

Chengcheng Zhong[1,2], Neil A. Smith[2], Daai Zhang[2], Simon Goodfellow[3], Ren Zhang[3], Weixing Shan[1,3], Ming-Bo Wang[2]

Supplementary File

Sequence of hpGUS-1 (The red letters represent the inverted repeat regions that form dsRNA stem. The black letters are the GUS downstream region that forms the loop of the hairpin RNA)

CCATGgTACGTCCTGTAGAAACCCCAACCCGTGAAATCAAAAAACTCGACGGCCTGTGGGCATTCAGTCTGGATC
GCGAAAACTGTGGAATTGATCAGCGTTGGTGGGAAAGCGCGTTACAAGAAAGCCGGGCAATTGCTGTGCCAGGCA
GTTTTAACGATCAGTTCGCCGATGCAGATATTCGTAATTATGCGGGCAACGTCTGGTATCAGCGCGAAGTCTTTA
TACCGAAAGGTTGGGCAGGCCAGCGTATCGTGCTGCGTTTCGATGCGGTCACTCATTACGGCAAAGTGTGGGTCA
ATAATCAGGAAGTGATGGAGCATCAGGGCGGCTATACGCCATTTGAAGCCGATGTCACGCCGTATGTTATTGCCG
GGAAAAGTGTACGTATCACCGTTTGTGTGAACAACGAACTGAACTGGCAGACTATCCCGCCGGGAATGGTGATTA
CCGACGAAAACGGCAAGAAAAAGCAGTCTTACTTCCATGATTTCTTTAACTATGCCGGAATCCATCGCAGCGTAA
TGCTCTACACCACGCCGAACACCTGGGTGGACGATATCTACCCGCTTCGCGTCGGCATCCGGTCAGTGGCAGTGA
AGGGCGAACAGTTCCTGATTAACCACAAACCGTTCTACTTTACTGGCTTTGGTCGTCATGAAGATGCGGACTTGC
GTGGCAAAGGATTCGATAACGTGCTGATGGTGCACGACCACGCATTAATGGACTGGATTGGGGCCAACTCCTACC
GTACCTCGCATTACCCTTACGCTGAAGAGATGCTCGACTGGGCAGATGAACATGGCATCGTGGTGATTGATGAAA
CTGCTGCTGTCGGCTTTAACCTCTCTTTAGGCATTGGTTTCGAAGCGGGCAACAAGCCGAAAGAACTGTACAGCG
AAGAGGCAGTCAACGGGGAAACTCAGCAAGCGCACTTACAGGCGATTAAAGAGCTGATAGCGCGTGACAAAAACC
ACCCAAGCGTGGTGATGTGGAGTATTGCCAACGAACCGGATACCCGTCCGCAAGGTGCACGGGAATATTTCGCGC
CACTGGCGGAAGCAACGCGTAAACTCGACCCGACGCGTCCGATCACCTGCGTCAATGTAATGTTCTGCGACGCTC
ACACCGATACCATCAGCGATCTCTTTGATGTGCTGTGCCTGAACCGTTATTACGGATGGTATGTCCAAAGCGGCG
ATTTGGAAACGGCAGAGAAGGTACTGGAAAAAGAACTTCTGGCCTGGCAGGAGAAACTGCATCAGCCGATTATCA
TCACCGAATACGGCGTGGATACGTTAGCCGGGCTGCACTCAATGTACACCGACATGTGGAGTGAAGAGTATCAGT
GTGCATGGCTGGATATGTATCACCGCGTCTTTGATCGCGTCAGCGCCGTCGTCGGTGAACAGGTATGGAATTTCG
CCGATTTTGCGACCTCGCAAGGCATATTGCGCGTTGGCGGTAACAAGAAAGGGATCTTCACTCGCGACCGCAAAC
CGAAGTCGGCGGCTTTTCTGCTGCAAAAACGCTGGACTGGCATGAACTTCGGTGAAAAACCGCAGCAGGGAGGCA
AACAATGAATCAACAACTCTCCTGGCGCACCATCGTCGGCTACAGCCTCGGGAATTAACAGACGCGTGGTTACAG
TCTTGCGCGACATGCGTCACCACGGTGATATCGTCCACCCAGGTGTTCGGCGTGGTGTAGAGCATTACGCTGCGA
TGGATTCCGGCATAGTTAAAGAAATCATGGAAGTAAGACTGCTTTTTCTTGCCGTTTTCGTCGGTAATCACCATT
CCCGGCGGGATAGTCTGCCAGTTCAGTTCGTTGTTCACACAAACGGTGATACGTACACTTTTCCCGGCAATAACA
TACGGCGTGACATCGGCTTCAAATGGCGTATAGCCGCCCTGATGCTCCATCACTTCCTGATTATTGACCCACACT
TTGCCGTAATGAGTGACCGCATCGAAACGCAGCACGATACGCTGGCCTGCCCAACCTTTCGGTATAAAGACTTCG
CGCTGATACCAGACGTTGCCCGCATAATTACGAATATCTGCATCGGCGAACTGATCGTTAAAACTGCCTGGCACA
GCAATTGCCCGGCTTTCTTGTAACGCGCTTTCCCACCAACGCTGATCAATTCCACAGTTTTCGCGATCCAGACTG
AATGCCCACAGGCCGTCGAGTTTTTTGATTTCACGGGTTGGGGTTTCTACAGGACGTAcCATGG

Sequence of hpGUS-2

GCCGTATGTTATTGCCGGGAAAAGTGTACGTATCACCGTTTGTGTGAACAACGAACTGAACTGGCAGACTATCCC
GCCGGGAATGGTGATTACCGACGAAAACGGCAAGAAAAAGCAGTCTTACTTCCATGATTTCTTTAACTATGCCGG
AATCCATCGCAGCGTAATGCTCTACACCACGCCGAACACCTGGGTGGACGATATCACCGTGGTGACGCATGTCGC
GCAAGACTGTAACCACGCGTCTGTTGACTGGCAGGTGGTGGCCAATGGTGATGTCAGCGTTGAACTGCGTGATGC
GGATCAACAGGTGGTTGCAACTGGACAAGGCACTAGCGGGACTTTGCAAGTGGTGAATCCGCACCTCTGGCAACC
GGGTGAAGGTTATCTCTATGAACTGTGCGTCACAGCCAAAAGCCAGACAGAGTGTGATATCTACCCGCTTCGCGT
CGGCATCCGGTCAGTGGCAGTGAAGGGCGAACAGTTCCTGATTAACCACAAACCGTTCTACTTTACTGGCTTTGG

TCGTCATGAAGATGCGGACTTGCGTGGCAAAGGATTCGATAACGTGCTGATGGTGCACGACCACGCATTTAATGG
ACTGGATTGGGGCCAACTCCTACCGTACCTCGCATTACCCTTACGCTGAAGAGATGCTCGACTGGGCAGATGAAC
ATGGCATCGTGGTGATTGATGAAACTGCTGCTGTCGGCTTTAACCTCTCTTTAGGCATTGGTTTCGAAGCGGGCA
ACAAGCCGAAAGAACTGTACAGCGAAGAGGCAGTCAACGGGGAAACTCAGCAAGCGCACTTACAGGCGATTCCAT
GGCGGCCGCGGGAATTCGATTGTGGTTAATCAGGAACTGTTGGCCCTTCACTGCCACTGACCGGATGCCGACGCG
AAGCGGGTAGATATCACACTCTGTCTGGCTTTTGGCTGTGACGCACAGTTCATAGAGATAACCTTCACCCGGTTG
CCAGAGGTGCGGATTCACCACTTGCAAAGTCCCGCTAGCGCCTTGTCCAGTTGCAACCACCTGTTGATCCGCATC
ACGCAGTTCAACGCTGACATCACCATTGGCCACCACCTGCCAGTCAACAGACGCGTGGTTACAGTCTTGCGCGAC
ATGCGTCACCACGGTGATATCGTCCACCCAGGTGTTCGGCGTGGTGTAGAGCATTACGCTGCGATGGATTCCGGC
ATAGTTAAAGAAATCATGGAAGTAAGACTGCTTTTTCTCGCCGTTTTCGTCGGTAATCACCATTCCCGGCGGGAT
AGTCTGCCAGTTCAGTTCGTTGTTCACACAAACGGTGATACGTACACTTTTCCCGGCAATAACATACGGC

Sequence of hpGFP

GGCTAGCTAATACGACTCACTATAGGGCAGCAGCACGGGGCCGTCGCCGATGGGGGTGTTCTGCTGGTAGTGGTC
GGCGAGCTGCACGCTGCCGTCCTCGATGTTGTGGCGGATCTTGAAGTTCACCTTGATGCCGTTCTTCTGCTTGTC
GGCCATGATGTATACGTTGTGGCTGTTGAAGTTGTACTCCAGCTTGTGCCCCAGGATGTTGCCGTCCTCCTTGAA
GTCGATGCCCTTCAGCTCGATGCGGTTCACCAGGGTGTCGCCCTCGAACTTCACCTCGGCGCGGGTCTTGTAGTT
GCCGTCGTCCTTGAAGAAGATGGTGCGCTCCTGGACGTAGCCTTCGGGCATGGCGGACTTGAAGAAGTCGTGCTG
CTTCATGTGGTCGGGGTAGCGGCTGAAGCACTGCACGCCGTAAGCGAAGGTGGTCACTAGTGTGGGCCAGGGCAC
GGGCAGCTTGCCGGTGGTGCAGATGAACTTCAGGGTCTAGACCGCGTCGGCATCCGGTCAGTGGCAGTGAAGGGC
GAACAGTTCCTGATTAGGGGGATGAAGCTACCTGGTCCGAACCACAAACCGTTCTACTTTACTGGCTTTGGTCGT
CATGAAGATGCGGACTTGCGTGGCAAAGGATTCGATAACGTGCTGATGGTGCACGACCACGCATTAATGGACTTT
ACCTTTTAATGGGGAATGAAGCTACCTGGTCCGAACTCCTACCGTACCTCGCATTACCCTTACGCTGAAGAGATG
CTCGACTGGGCAGATGAACATGGCATCGTATTTAGGTGACACTATAGCCCTGAAGTTCATCTGCACCACCGGCAA
GCTGCCCGTGCCCTGGCCCACACTAGTGACCACCTTCGCTTACGGCGTGCAGTGCTTCAGCCGCTACCCCGACCA
CATGAAGCAGCACGACTTCTTCAAGTCCGCCATGCCCGAAGGCTACGTCCAGGAGCGCACCATCTTCTTCAAGGA
CGACGGCAACTACAAGACCCGCGCCGAGGTGAAGTTCGAGGGCGACACCCTGGTGAACCGCATCGAGCTGAAGGG
CATCGACTTCAAGGAGGACGGCAACATCCTGGGGCACAAGCTGGAGTACAACTTCAACAGCCACAACGTATACAT
CATGGCCGACAAGCAGAAGAACGGCATCAAGGTGAACTTCAAGATCCGCCACAACATCGAGGACGGCAGCGTGCA
GCTCGCCGACCACTACCAGCAGAACACCCCCATCGGCGACGGCCCCGTGCTGCTGCCGTCGACC

Primers used for RT-PCR analysis of dsRNA stem and loop of hpGUS-1

Primer	Sequence	Use
GUS-as-R	5′CCTGTAGAAACCCCAACCCG	For reverse transcription
qPCR-STEM-F	5′GACATCGGCTTCAAATGGCG	qPCR
qPCR-STEM-R	5′TGGATCGCGAAAACTGTGGA	qPCR
qPCR-LOOP-F	5′AGCAGGGAGGCAAACAATGA	qPCR
qPCR-LOOP-R	5′AGACTGTAACCACGCGTCTG	qPCR

Plasmid Maps

pMBW306

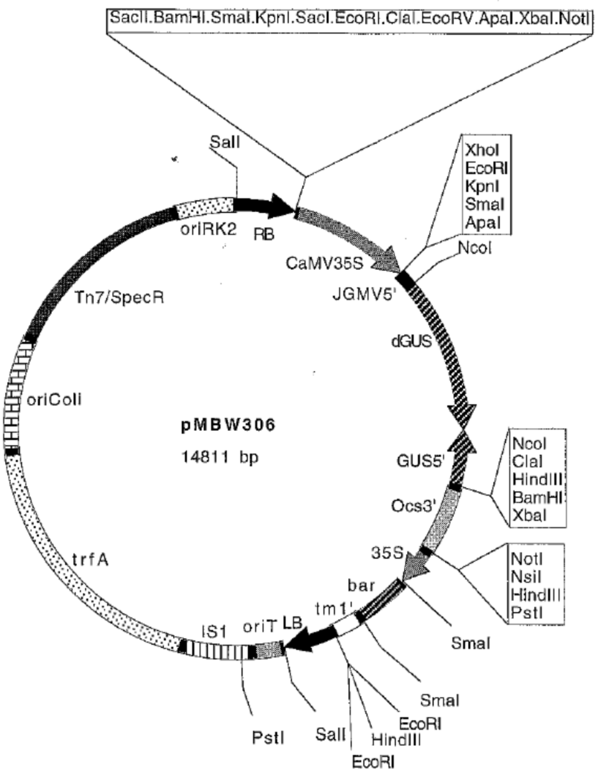

A rough restriction map for pMBW306 encoding hpGUS-1. The GUS inverted repeat sequence plus the 5' Johnson Mosaic Virus (JGMV) sequence of the GUS hpRNA construct in Wang et al [1] was cloned into pART7 [2] and the 35S-hpGUS-OCS3' cassette was then cloned into pWBVec4a [2].

pMBW281

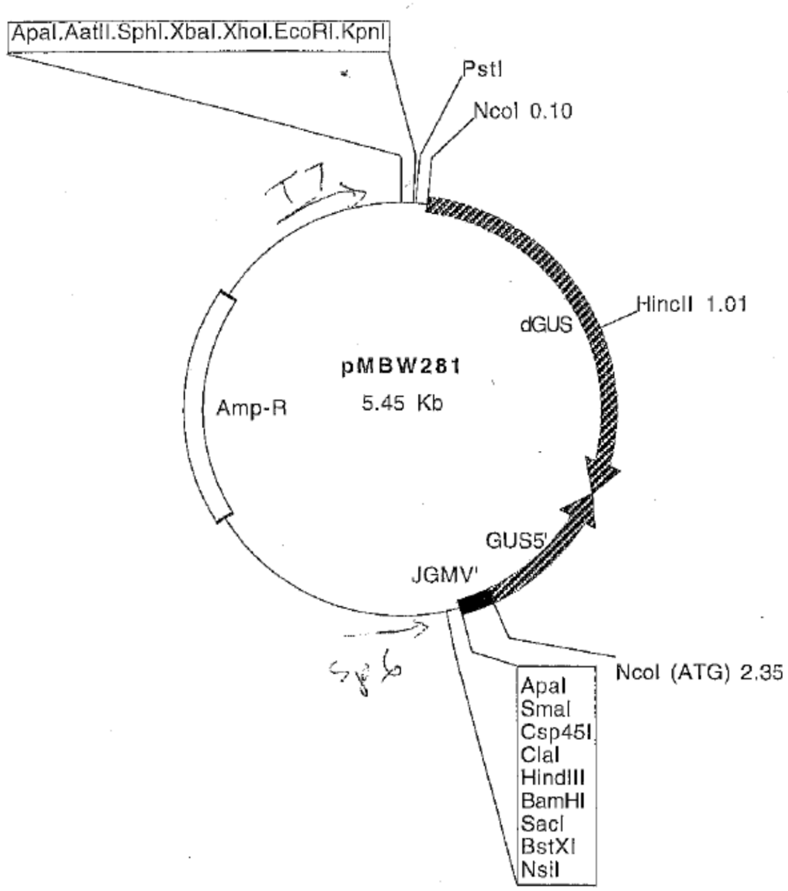

A rough restriction map for pMBW281 encoding hpGUS-1. The GUS inverted repeat sequence plus the 5' Johnson Mosaic Virus (JGMV) sequence of the GUS hpRNA construct in Wang et al [1] was cloned in pGEM-7Z (Promega).

pBact-term-JKK

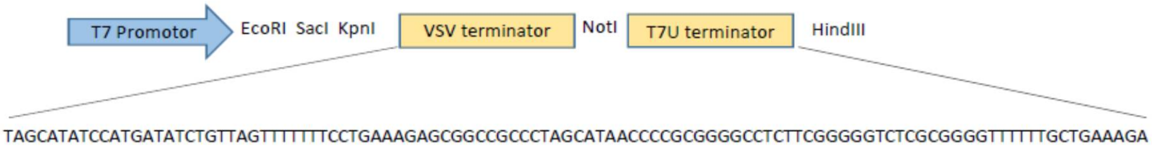

A schematic of the T7 promoter-terminator cassette pBact-term-JKK. The terminator sequence was based on Mairhofer et al [4]. The cassette was built in the plasmid pJKK, which was made by replacing the ampicillin resistance gene in pGEM 3Zf(-) (Promega) with the kanamycin resistance gene.

References

1. Wang, M.B.; Waterhouse, P.M. High-efficiency silencing of a b-glucuronidase gene in rice is correlated with repetitive transgene structure but is independent of DNA methylation. Plant Mol. Biol. **2000**, *43*, 67–82.
2. Gleave, A.P. A versatile binary vector system with a T-DNA organisational structure conducive to efficient integration of cloned DNA into the plant genome. *Plant Mol. Biol*. **1992**, *20*, 1203–1207.
3. Wang, M.B.; Li, Z.Y.; Matthews, P.R.; Upadhyaya, N.M.; Waterhouse, P.M. Improved vectors for *Agrobacterium tumefaciens*-mediated transformation of monocot plants. *Acta Horticulturae* **1998**, *461*, 401-407.
4. Mairhofer, J.; Wittwer, A.; Cserjan-Puschmann, M.; Striedner, G. Preventing T7 RNA polymerase read-through transcription-A synthetic termination signal capable of improving bioprocess stability. *ACS Synth Biol*. **2015**, *4*, 265-73.
